# Design and validation of a semi-quantitative microneutralization assay for human Metapneumovirus A1 and B1 subtypes

**DOI:** 10.1038/s41598-025-96567-6

**Published:** 2025-04-04

**Authors:** Giulia Riolo, Valentina Biagini, Noemi Guerrini, Giulia Roscia, Roberta Antonelli, Ginevra Giglioli, Maria Alfreda Stincarelli, Pietro Piu, Carolina Bonifazi, Simona De Grazia, Mariangela Pizzo, Piero Lovreglio, Angela Stufano, Claudia Maria Trombetta, Alessandro Manenti, Emanuele Montomoli, Francesca Dapporto

**Affiliations:** 1 VisMederi S.R.L., Siena, Italy; 2 Department of Life Sciences, University of Siena, Siena, Italy; 3 Department of Health Promotion, Mother and Child Care, Internal Medicine and Medical Specialties “G. D’Alessandro”, University of Palermo, Palermo, Italy; 4 Interdisciplinary Department of Medicine, Section of Occupational Medicine, University of Bari, Bari, Italy; 5 Department of Molecular and Developmental Medicine, University of Siena, Siena, Italy; 6 VaepiX, Joint Research Laboratory, University of Siena, Siena, Italy

**Keywords:** ELISA, Infectious diseases

## Abstract

Since 2001, human Metapneumovirus has been a significant cause of human respiratory disease worldwide, and no vaccine or preventive treatment is currently available. The ELISA-based live virus microneutralization assay is a method to detect neutralizing antibodies against a target pathogen. The aim of this study was to demonstrate the suitability of this approach to quantifying neutralizing antibodies against A1 and B1 virus subtypes in human serum samples. To standardize and validate this microneutralization assay, we carried out analytical procedures according to the International Council of Harmonization guidelines; these procedures are described in detail. In addition, we compared the validated method with the indirect ELISA, and confirmed that the ELISA-based microneutralization assay provides reliable, accurate and reproducible results. The use of this high-throughput method for large-scale serological studies could effectively support the evaluation of the immunogenicity of new vaccines, thereby improving therapeutical strategies against human Metapneumovirus.

## Introduction

### Human Metapneumovirus from discovery to virus biology

First discovered in 2001 in the Netherlands by van den Hoogen^1^, human Metapneumovirus (hMPV) is a lipid-enveloped virus with negative-sense, single-stranded RNA, and belongs to the *Pneumoviridae* family and *Metapneumovirus* genus. Worldwide, two genetic lineages (A and B) of hMPV are circulating that are antigenically distinct and can each be further divided into genetic sublineages: A1 (NL/1/00), A2 (NL/17/00), B1 (NL/1/99) and B2 (NL/1/94)^2^. To date, all subtypes have been evenly found across the globe with no differences in pathogenicity and no predominance of one subtype over another.

Knowledge on the pathogenic mechanism induced by hMPV is still unclear. Phylogenetic analysis has revealed that genetic diversity among subtypes is mainly due to variability in sequences encoding structural external proteins (Fusion, Glycoprotein and Small Hydrophobic protein), which play a crucial role in cell infection^3–6^. The Fusion (F) protein is the best characterized among surface hMPV proteins. It belongs to class I viral glycoprotein and is expressed as a homotrimer on the viral envelope. The protein is first synthesized as a precursor (F0). After proteolytic cleavage, the trimer becomes a functional pre-fusion (Pre-F) polypeptide; in this active state, Pre-F undergoes conformational changes that allow the protein to turn into a more stable post-fusion (Post-F) conformation. This rearrangement is crucial for virus attachment to receptors on cell surface, following virus-cell membranes fusion^7^. Unlike the F protein, the Glycoprotein G can only promote virus attachment to the cell, but it is not able to initiate infection on its own. However, it plays a key role as an immune response modulator inhibiting several cellular pathways, such as Interferon Type 1, in order to enhance viral infection. The Small Hydrophobic (SH) protein is also involved in this process, as it acts as a viroporin, increasing membrane permeability to facilitate virus entry^2–7^.

Interestingly, the G protein shows high variability (30–37% sequence identity) among virus subtypes, whereas the F protein is highly conserved (94–100% sequence identity) and shares 30% sequence homology with the human Respiratory Syncytial Virus (RSV) F protein^7^. Of note, F protein is the sole target of neutralizing antibodies (nAbs) to hMPV infection, whereas the antibody response elicited by G or SH proteins is not protective^4,6,8^. This is why the F protein is the most attractive target for vaccine development against hMPV.

### Epidemiology and state of art vaccine research

hMPV appears to have a worldwide distribution, being detected throughout the year with a lower impact during late spring, summer and fall. Generally, it has been shown that hMPV A and B subgroups usually circulate simultaneously each year, though specific strain diffusion varies from area to area, with local outbreaks^9^. hMPV spreads via droplets among population and infection involves both the upper and lower respiratory tract, causing fever, cough, pharyngitis, otitis, wheezing, asthma, hypoxia, bronchiolitis and pneumonia^7,9,10^. hMPV primarily affects infants and young children, with > 90% of individuals scoring positive for hMPV infection by the age of 5 years. Of note, a seroprevalence of > 90% is observed in infants aged less than 3 months, reflecting the presence of maternally derived antibodies. This percentage reaches 100% by adulthood. In young children, infection frequently causes asthma, requiring hospitalization if acute respiratory failure occurs. In adults, infection is mild, but it is complicated by the coexistence of factors such as immunodeficiency or underlying respiratory diseases^9–11^.

Although no vaccine against hMPV has been licensed, several studies in animal models have shown that live recombinant parainfluenza viruses expressing the hMPV F protein can induce immunogenicity and the production of hMPV-specific nAbs^9,11^. A similar outcome was observed when attenuated recombinant viruses lacking G, SH or M2 genes were used^12,13^. Another study highlighted the possibility to produce a recombinant attenuated vaccine by replacing N or P hMPV genes with their avian pneumovirus counterparts^9^. In addition to live-attenuated vaccines (LAVs), approaches involving the use of virus-like particles vaccines (VLPVs) and monoclonal antibodies (mAbs) have been investigated^11–13^. Regarding VLPVs, one study combined retroviral proteins to form a structural core that displayed F and G proteins from hMPV on its surface; when this vaccine was injected into mice, it induced a massive humoral response and cross-protective immunization^12,13^. As in the case of the licensed anti-RSV Palivizumab, strategies involving the production of mAbs against the hMPV F protein, mainly as a prophylactic measure, have proved effective in animal models, paving the way for their use also in clinical trials^7^.

### Sero-epidemiology for vaccine assessment

hMPV sero-epidemiology has been investigated by implementing various types of serological assays. Enzyme-linked immunosorbent assays (ELISA) that use recombinant F and N proteins or whole purified virions have been developed, and have proved to be effective and sensitive in detecting hMPV-specific antibodies^14–16^. Cell-based assays that use both the recombinant F protein and live hMPV as source of infection, and immunofluorescence as readout, have also been employed^17^. These assays have the advantage of detecting nAbs; however, the readout used is highly subjective and cannot be applied to large-scale serological studies due to its low throughput.

### Aim of the study

The aim of this study was to demonstrate the suitability of a high-throughput ELISA-based microneutralization assay (EMN) for detecting nAbs against hMPV-A1 and hMPV-B1 subtypes in human serum samples. The method was validated according to the guidelines of the International Council for Harmonization (ICH), in order to overcome the main drawbacks of common serological assays. The EMN shows the potential for higher throughput than traditional methods, enabling large-scale screening of samples to evaluate immunogenicity of new vaccines.

## Results

### Virus propagation

**Fig. 1:**
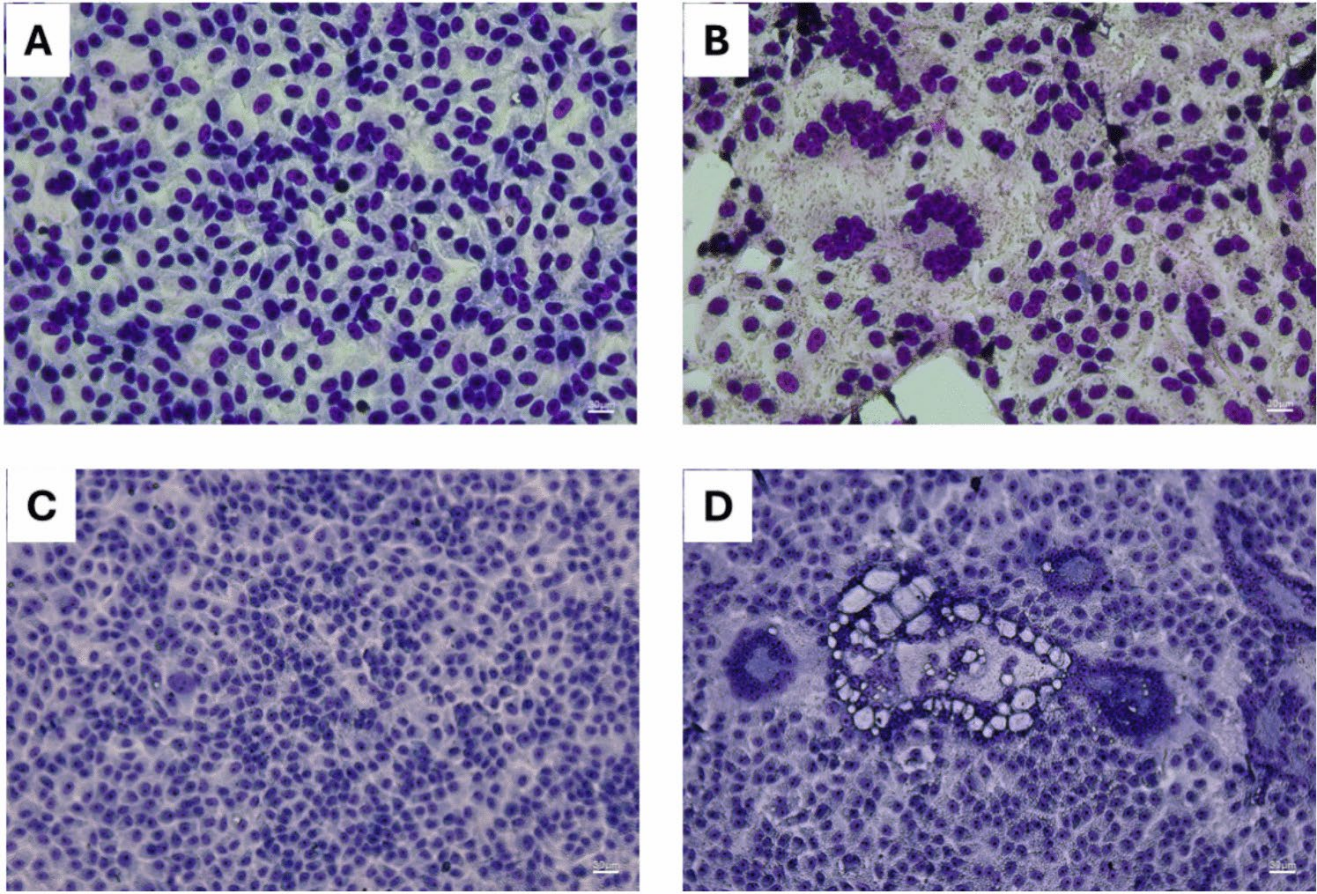
Syncytium induced by virus infection in Vero E6 cell line. Uninfected cells (**A** and **C**) were compared with cells infected by hMPV-A1 (**B**) and hMPV-B1 (**D**), respectively. Cells were stained by means of the Fast staining in haematology (fast panoptic) kit (PanReac AppliChem). Images were acquired with a Leica Mateo TL microscope.

Few published data are available on hMPV in vitro cultivation and its replication abilities^18^. Our virus propagation set-up used Vero E6, a Vero cell clone particularly suited to growing viruses that replicate slowly. In addition, this cell line is tolerant of trypsin, a feature that is crucial to mimicking in vitro the proteolytic activation of the F protein, which promotes virus infection. As shown in Fig. 1, Vero E6 are sensitive to hMPV infection.

### Set-up of the ELISA-based microneutralization assay

A series of preliminary analyses was performed for the purpose of designing the EMN assay. To this end, both Vero E6 cell suspension and cell adhesion were tested; the EMN incubation times of 24, 48 and 72 h were investigated; finally, a cross-test was performed to find the best primary and secondary antibody concentrations. The optimal conditions consisted of Vero E6 seeding at a concentration of 2 × 10^5^ cell ml^−1^ 24 h before the test; the use of DMEM supplemented with 1% FBS and 3 µg ml^−1^ of trypsin and the use of a 1:1000 dilution ratio for primary and secondary antibodies. The best incubation time of hMPV-A1 was 24 h, while that of hMPV-B1 was 48 h.

**Fig. 2:**
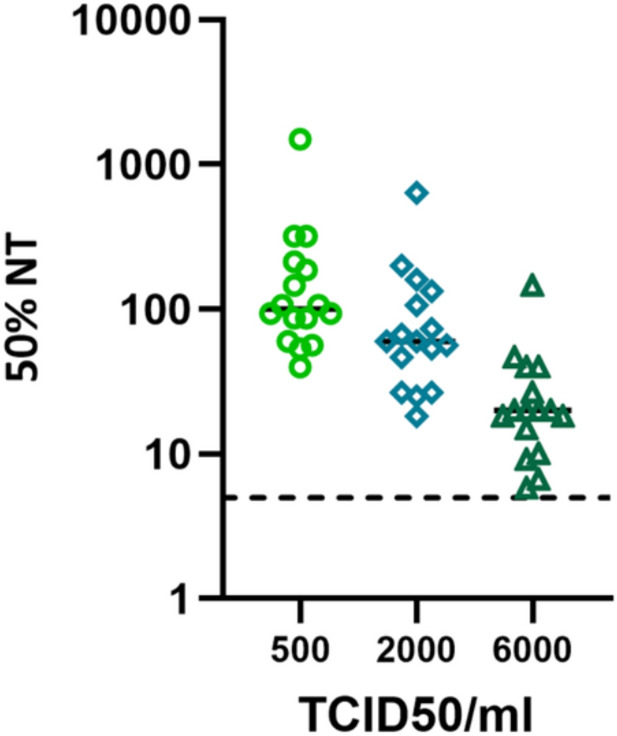
Viral dose selection and evaluation of the impact on nAb titers. A total of 16 samples were tested. Each dot/square/triangle represents the geometric mean titer (GMT) retrieved from three independent tests of a single serum sample tested in duplicate in each test at different viral doses (500, 2000 and 6000 TCID50 ml^−1^) for hMPV-A1; the *y axis* shows 50% neutralizing titer (NT) expressed as the reciprocal of the serum dilution. The lower limit of detection was set at 5, which is half of the reciprocal of the first serum dilution in plates (dashed black line). Cut-off for the calculation of the 50% NT was determined by applying the formula: *cut-off* = *((average of VC wells- average of CC wells)/2)* + *average of CC wells*. CC: cell control; VC: viral control.

After the experimental design of the method had been established, 16 commercially available samples were screened to evaluate the sensitivity of the assay. Samples were tested by using hMPV-A1 virus at three different concentrations: 500 TCID50 ml^−1^, 2000 TCID50 ml^−1^ and 6000 TCID50 ml^−1^. Three independent runs for each dose were performed, on three different days. All tests performed yielded similar results (Supplementary Fig. 1), thus demonstrating the high reproducibility of the assay, but more importantly we found that the nAb titers of the tested samples were significantly viral dose-dependent. The optimal infective dose to be used was demonstrated to be 2000 TCID50 ml^−1^, since this was the condition in which the assay showed the best balance between sensitivity and specificity. Results are shown in Fig. 2.

### Validation of the ELISA-based microneutralization assay

#### Dilutional linearity

Linearity is assessed to determine the integrity of the sample dilution procedure. A given sample of interest with a high concentration above the upper limit of quantitation can be diluted to a concentration within the range of the dilution factors evaluated during validation and still give a reliable result^19^.

**Fig. 3:**
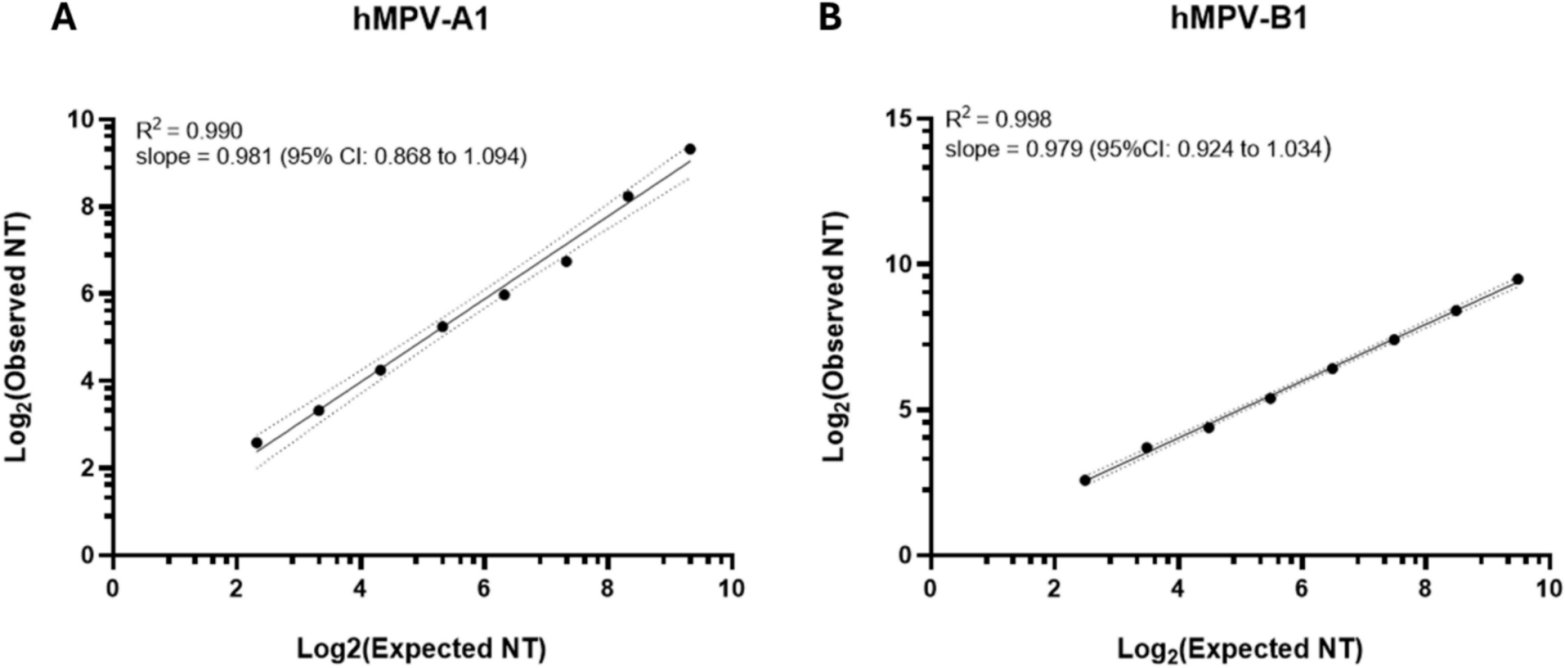
Dilutional linearity. Data for hMPV-A1 (**A**) and hMPV-B1 (**B**) show log_2_ of the expected GMT on the *x-axis* and log_2_ of the observed GMT on the *y-axis*. Dotted black lines represent the upper and lower limits of 95% CI. GMT: geometric mean titer; CI: confidence interval; NT: neutralizing titer.

Our results met all acceptance criteria for linearity in the EMN assay if the 95% CI (Confidence Interval) of the slope was between 0.7 and 1.3. Data obtained in linearity experiments are shown in Supplementary Table 1, while the coefficient of determination (R^2^) and the absolute values of the slope obtained in our study are reported in Fig. 3.

#### Relative accuracy

**Fig. 4:**
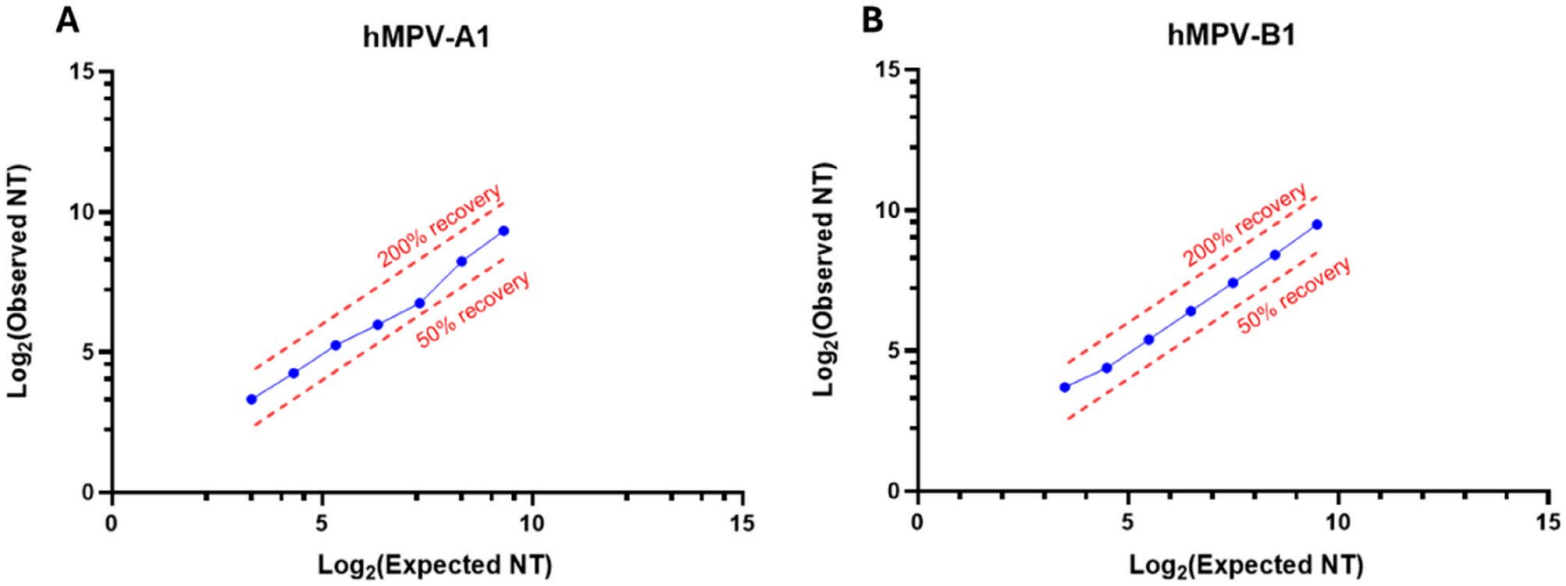
Relative accuracy. Data for hMPV-A1 (**A**) and hMPV-B1 (**B**) show log_2_ of the expected GMT on the *x-axis* and log_2_ of the observed GMT on the *y-axis*. Relative accuracy was evaluated by calculating the percentage of recovery on the GMT of the observed values and the expected (true) titers obtained by applying the following formula: 100*(observed GMT/expected GMT). Dashed red lines represent 50% and 200% recovery of the expected titer. GMT: geometric mean titer; NT: neutralizing titer.

This parameter is assessed as the agreement between the expected and the observed titers across the dilution series. The assay is considered to have acceptable relative accuracy if the observed GMT of all replicates obtained for a sample is within 50–200% of the expected titer. Considering that when there is no difference between observed and expected GMT, the ratio corresponds to 100%, the acceptable level of variability is defined such that the observed GMT result for any sample should not differ by more than ± twofold from the expected GMT. The EMN assay proved accurate from 1:1 to 1:64 dilution for both hMPV strains, as depicted in Fig. 4 and reported in Supplementary Table 2.

#### Precision

Precision is usually expressed by the variance, standard deviation or coefficient of variation of a series of measurements^20^. The variability between results obtained from independent tests can be assessed by evaluating three different conditions: Repeatability, Intermediate precision and Format Variability (FV).

Repeatability is considered to have acceptable intra-run precision if all samples display a geometric coefficient of variation (GCV) ≤ 65.5%.

Intermediate precision is determined across two operators in different runs. The assay is deemed to be precise if all estimates of intermediate precision display GCV ≤ 129.0%.

In our study, the EMN assay proved repeatable and precise from 1:1 up to 1:64 dilution for both HMPV strains.

The assay is considered to have acceptable FV if all samples display GCV ≤ 81.5%; this criterion was met for both subtypes.

Results are summarized in Table 1, while how to calculate the GCV, intermediate precision and FV is explained in Supplementary Data Analysis 1.

**Table 1:** Repeatability, Intermediate precision and Format Variability data for hMPV-A1 and hMPV-B1. Values expressed in percentage indicate GCV. Acceptability criteria: GCV ≤ 65.5% for Repeatability; GCV ≤ 129.0% for Intermediate precision; GCV ≤ 81.5% for Format Variability. The assay meets all criteria for both strains. GCV: geometric coefficient of variation; GMT: geometric mean titer.

hMPV-A1								
Fold dilution	Observed GMT	Repeatability (%)	Fold dilution	Observed GMT	Intermediate precision (%)	Fold dilution	Observed GMT	Format variability (%)
1	640	0.0%	1	640	0.0%	1	640	0.0%
2	302	22.2%	2	302	22.2%	2	302	15.2%
4	107	41.4%	4	107	43.2%	4	107	28.9%
8	63	39.8%	8	63	41.4%	8	63	27.8%
16	38	22.2%	16	38	22.2%	16	38	15.2%
32	19	22.2%	32	19	22.2%	32	19	15.2%
64	10	0.0%	64	10	0.0%	64	10	0.0%

hMPV-B1								
Fold dilution	Observed GMT	Repeatability (%)	Fold dilution	Observed GMT	Intermediate precision (%)	Fold dilution	Observed GMT	Format variability (%)
1	718	22.2%	1	718	56.4%	1	718	37.2%
2	339	22.2%	2	339	22.2%	2	339	15.2%
4	170	22.2%	4	170	22.2%	4	170	15.2%
8	85	41.4%	8	85	43.2%	8	85	28.9%
16	42	22.2%	16	42	22.2%	16	42	15.2%
32	21	32.7%	32	21	46.7%	32	21	31.1%
64	13	22.2%	64	13	46.7%	64	13	31.1%

#### Limit of quantitation and range

From the results obtained, the Lower and Upper Limits of Quantitation (LLOQ and ULOQ, respectively) were calculated: the LLOQ was set at 10 and the ULOQ was set at 640 for hMPV-A1, while hMPV-B1 range was 10-1280.

#### Specificity

Specificity demonstrates the ability of the assay to detect and distinguish the analyte of interest from other analytes^20,21^. To assess this parameter, the positive sample for homologous virus must show a GMT with at least a four-fold difference from the heterologous samples tested. The negative sample must have a negative titer in all measurements of this parameter. Results shown in Table 2 and Supplementary Table 3 indicated that the method was specific for both hMPV strains.

**Table 2:** Specificity results showing fold-change for hMPV-A1 and hMPV-B1. Specificity samples: SS1) hMPV-positive sample by PCR; SS2) Influenza Anti-A/Victoria/2570/2019-like (H1N1) HA Serum 21/120; SS3) Influenza anti-A/Cambodia/e0826360/ 2020-Like (H3N2) HA Serum 21/118; SS4) Influenza anti-B/Washington/02/2019-like (B Victoria lineage) HA Serum 19/318; SS5) Influenza anti-B/Phuket/3073/2013-like HA serum (B Yamagata lineage) 19/322; SS6) Sun Diagnostic cod. INT-01H.

	SS1/SS2	SS1/SS3	SS1/SS4	SS1/SS5	SS1/SS6
hMPV-A1	128	128	128	128	128
hMPV-B1	128	128	128	256	256

#### Robustness

This parameter was evaluated for both virus strains testing the following two conditions: cell seeding concentration and incubation time of serum-virus mixture.

In the first condition, three different concentrations were tested for each subtype: the standard concentration (2.0 × 10^5^cell ml^−1^) and two non-standard concentrations (1.5 × 10^5^cell ml^−1^ and 2.5 × 10^5^cell ml^−1^). Positive and negative controls were tested in four replicates per plates, by two operators for each condition, thus obtaining 24 values for each analyte. The results obtained in each condition were aggregated to obtain 12 RP values for each sample. Regarding this condition, the GCV for the positive sample was 25.32% and 36.62% against hMPV-A1 and hMPV-B1, respectively.

Plates with serum-virus mixture were incubated for three different incubation times before the transfer on cell monolayer, to assess the influence of the virus incubation time on the assay: 60 min (standard condition), 45 min and 75 min. Samples were tested in the same way as for cell seeding, thus obtaining 24 values and 12 RP for each sample. The GCV for the positive sample was 24.04% for hMPV-A1 and 29.85% for hMPV-B1. All the above mentioned results met the acceptance criteria set at GCV ≤ 45%, indicating that the EMN method was robust (Supplementary Table 4).

### Evaluation of neutralizing titers in serum samples by the ELISA-based microneutralization assay

**Fig. 5:**
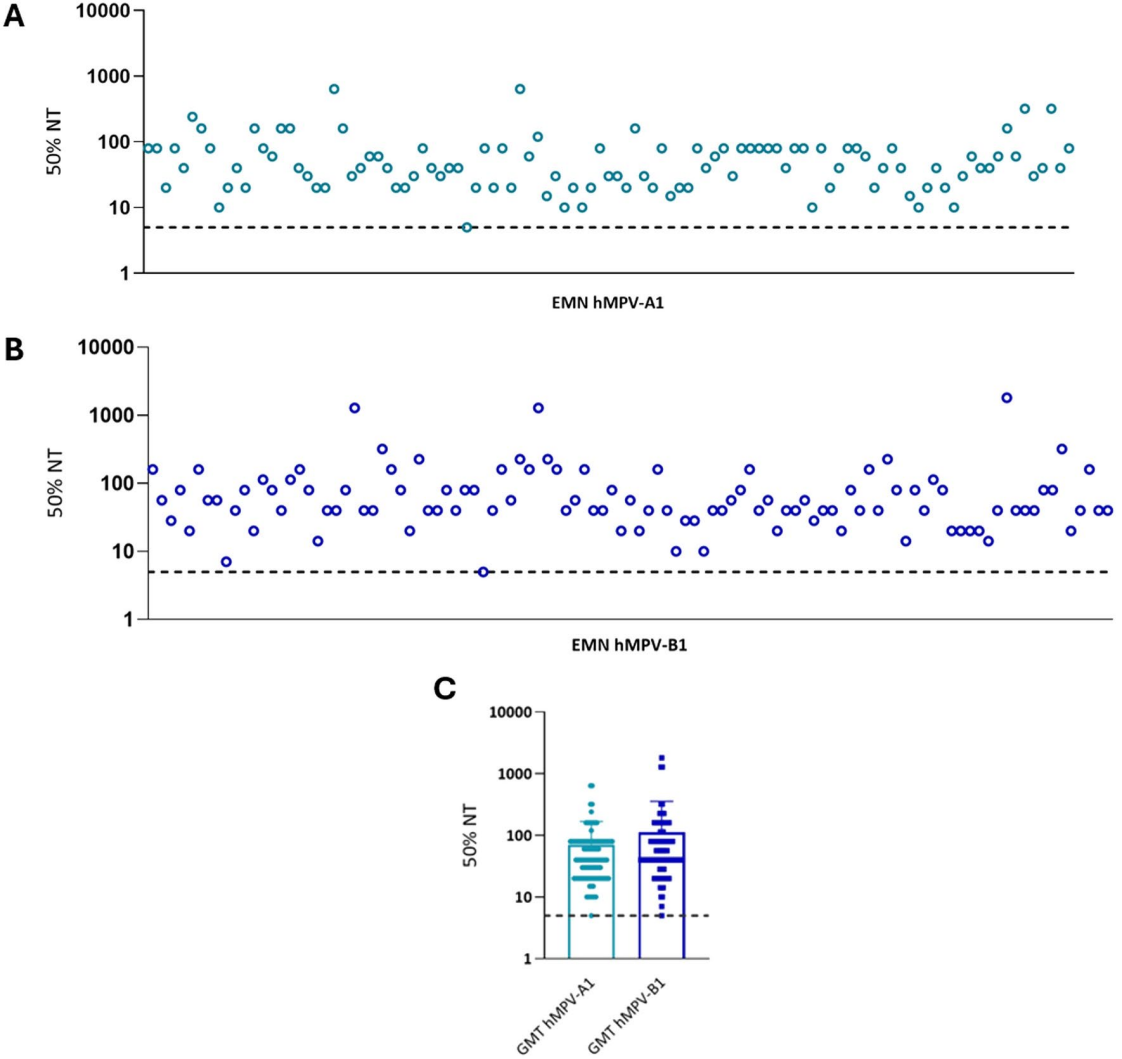
Quantification of neutralizing titers in human serum samples. Resulting data from EMN assay for hMPV-A1 (**A**) and hMPV-B1 (**B**), then cumulative results from both strains (**C**). Each dot/square represents the GMT of a single sample tested in duplicate; the *y axis* shows 50% NT expressed as the reciprocal of the serum dilution. The lower limit of detection was set at 5, which is half of the reciprocal of the first serum dilution in plates (dashed black line). Cut-off for the calculation of the 50% NT was determined as described in Fig. 2. GMT: geometric mean titer; NT: neutralizing titer.

Following the set-up and validation of the EMN assay, a cohort of 105 human serum samples was analyzed for the presence of nAbs both against hMPV-A1 and hMPV-B1 on using 2000 TCID50 ml^−1^ as the viral dose. The neutralizing titers shown in Fig. 5 indicate a marked response, mostly ranging from 20 to 160, for most samples of both strains. Of note, for those samples that did not lie within this range, when either a higher or a lower response to hMPV-A1 was observed, the same trend was also seen for hMPV-B1, reflecting the reliability of the assay and excluding the presence of outliers. Overall, these results indicate that a moderate humoral response was detected in the cohort examined and that this response was similar for both strains analyzed.

### Application of indirect ELISA to quantify binding antibody titers in serum samples

The same panel was then analyzed by means of an indirect ELISA, which allows for the detection of both neutralizing and non-neutralizing antibodies. Serum antibodies were tested for their binding to either hMPV-A1 or hMPV-B1 glycoprotein G and hMPV-B1 F0 glycoprotein only, since the homologous counterpart of hMPV-A1 F0 was not commercially available at the time of analysis.

**Fig. 6:**
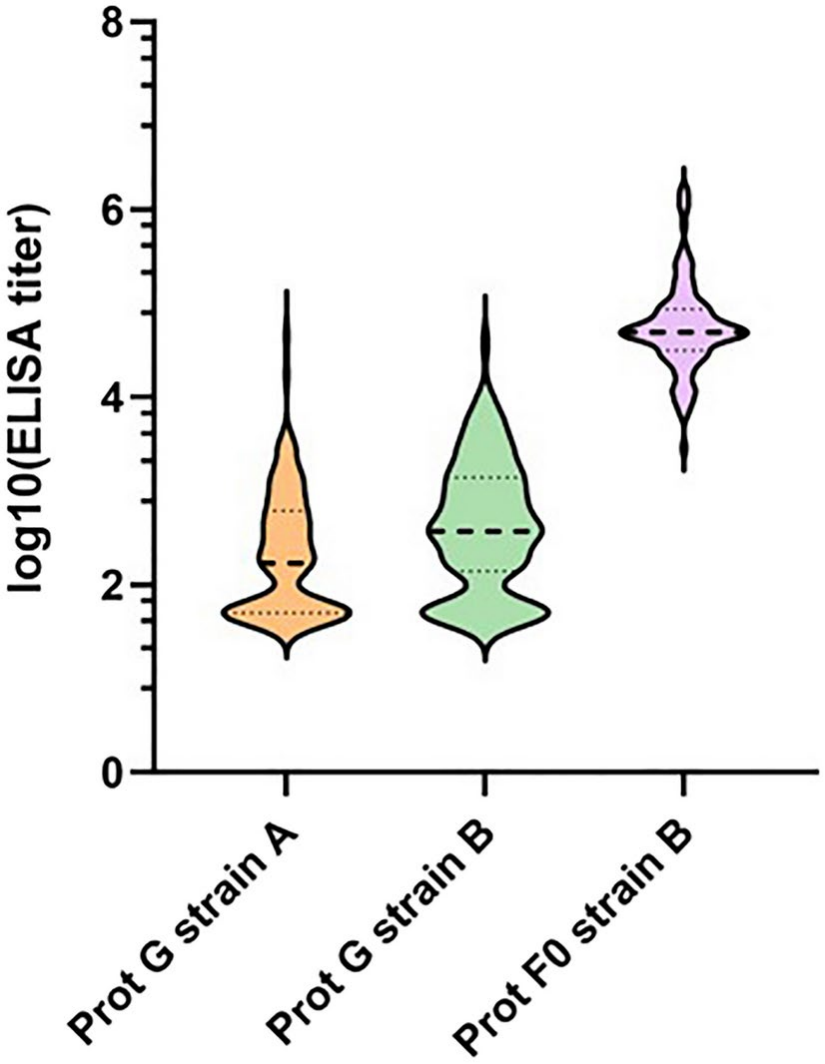
Quantification of neutralizing and non-neutralizing antibody titers through indirect ELISA. Cut-off was calculated by multiplying by three the average of blank OD signal derived from six micro-wells containing sample diluents and secondary HRP-antibody. IgG results were calculated by relating the OD value of each specimen to the respective cut-off value of the plate. Samples with ODs below the cut-off value were considered negative and the titer assigned in this case was half of the starting dilution. Samples in which ODs were above the cut-off value were deemed positive and a titer was calculated. Dashed bold black lines indicate medians and dashed thin black lines indicate quartiles.

As illustrated in Fig. 6, a similar low-to-modest response was registered for glycoprotein G for both strains, whereas a positive moderate-to-high response was recorded for hMPV-B1 glycoprotein F0. Taken together, these data confirm the presence of a humoral response against hMPV-A1 and hMPV-B1 in the panel tested, and suggest that glycoprotein F0 possesses a stronger immunogenicity than glycoprotein G for hMPV-B1, highlighting it as a preferable serological marker.

### Correlation of ELISA-based microneutralization assay and ELISA results

In order to evaluate the strength of the relationship between the data retrieved from the EMN assay and those obtained from indirect ELISA, Spearman’s correlation analysis was performed.

**Fig. 7:**
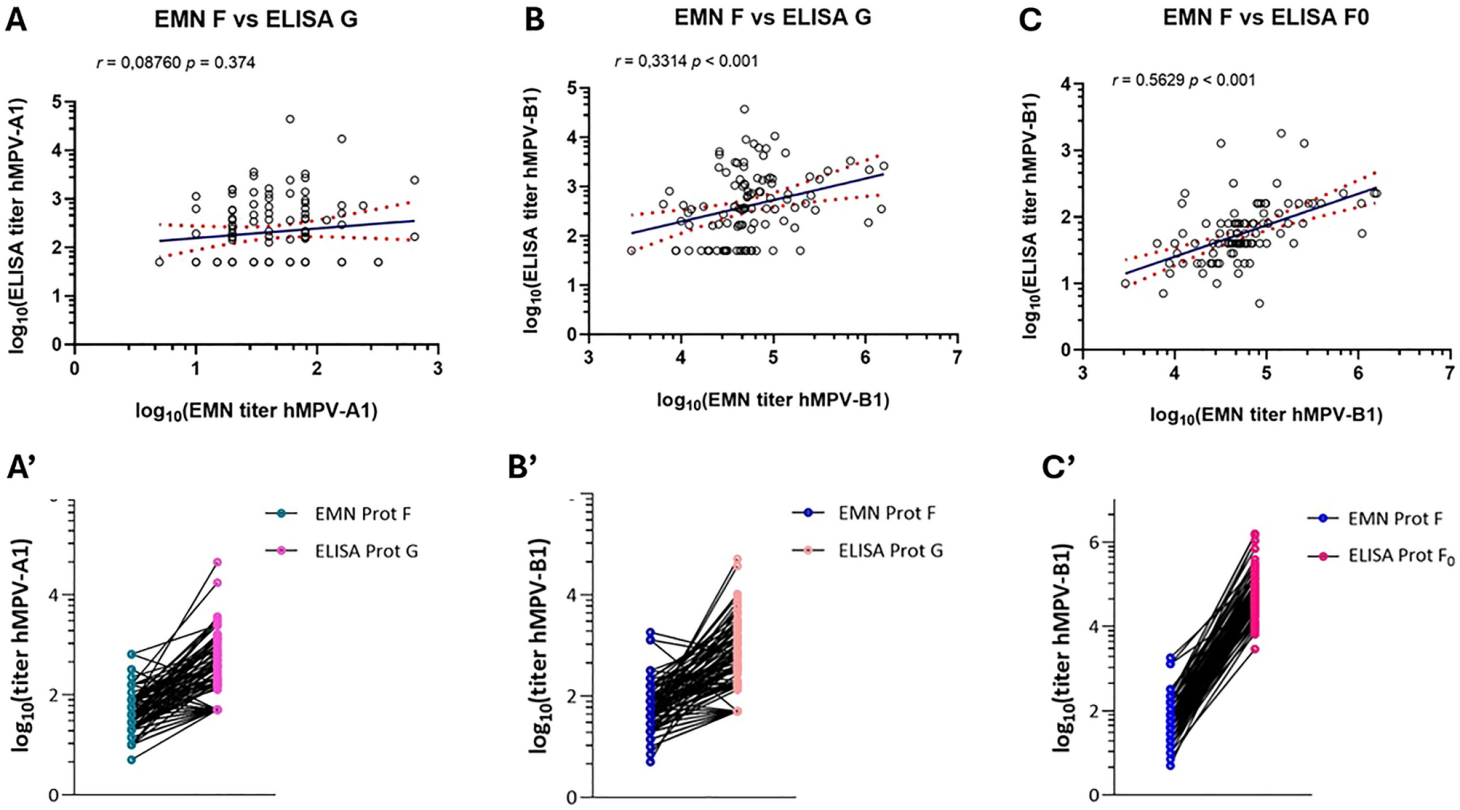
Correlation of EMN assay and ELISA results. Spearman correlation analysis of antibody titers expressed in log_10_ for hMPV-A1 EMN F protein versus hMPV-A1 ELISA G protein (**A**), for EMN F protein versus ELISA G protein hMPV-B1 (**B**) and for EMN F protein versus ELISA F0 protein hMPV-B1 (**C**). Blue line indicates the linear regression line and dashed red lines indicate 95% CI. Paired graphs (**A**′, **B**′ and **C**′) combine antibody titers derived from EMN assay and ELISA; each dot represents the GMT, expressed in log_10_, of a single serum sample, tested in duplicate for each methodology. CI: confidence interval; GMT: geometric mean titer.

First, antibody titers from EMN against F protein and ELISA against G glycoprotein were correlated. For both hMPV-A1 and hMPV-B1 strains, Spearman’s rho value (*r*) revealed a weak correlation, which was *r* = 0.0876 for hMPV-A1 and *r* = 0.3314 for hMPV-B1 (Fig. 7A and B). These results were corroborated when individual antibody titers for each method were plotted and connected by a straight line to analyze the trend of the association, as shown in Fig. 7A′ and B′.

By contrast, a significant, albeit moderate, correlation (*r* = 0.5829) emerged when we analyzed the relationship between neutralizing titers from the EMN against the F protein and IgG amounts from ELISA against the F0 glycoprotein for hMPV-B1 (Fig. 7C). The magnitude of the correlation observed can perhaps be explained by the fact that the ELISA assay detects antibodies against F0, which is the unstable precursor of the pre-F protein, while the EMN assay identifies antibodies against both pre-F and post-F conformations. Again, this stronger association was confirmed by plotting individual values, as shown in Fig. 7C’. Overall, these data suggest that the highest concordance between the two methodologies was observed on comparing serum antibody titers produced against the F protein, further validating its use as a marker in serological assays.

## Discussion

Since the discovery of hMPV in 2001, many advances have been made in understanding its mechanism of infection and the impact of this virus in humans. hMPV is a significant cause of human respiratory disease worldwide. Upper and lower tract infections can be more severe in young children, frequently requiring hospitalization; effective treatments are therefore needed. However, there is still much to be discovered, mainly regarding the pathogenesis of hMPV, and, notwithstanding 20 years of efforts, there is currently no specific therapy or prevention against hMPV.

As in the case of its family member hRSV, the traditional approach to vaccine development was based on virus inactivation by formalin, which produced a poor humoral response in animal models^22,23^. More recent vaccine design strategies have focused on developing an antibody-driven complex, such as the production of mAbs that target neutralizing viral antigen, which may block virus transmission. mAbs are appealing as a potential medical approach because of their high specificity and their ability to elicit immune responses by triggering antigen-specific humoral immunity against the target pathogen as a correlate of protection^24,25^. The F glycoprotein has been identified as the main immunogenic antigen for hMPV, because of its ability to elicit a significant response of serum nAbs. By contrast, differently from hRSV, the G protein induces a poorly protective response against hMPV and is therefore not considered an attractive target for vaccine assessment. Furthermore, F domains are highly conserved among all hMPV subtypes, which should facilitate the development of treatments with broad-spectrum activity.

In this context, the establishment of a reliable serological assay for the detection of nAbs against a target pathogen after natural infection and/or vaccination has a great positive implication for therapeutical strategies.

The aim of this study was to describe and validate an ELISA-based microneutralization assay (EMN) to measure nAb titers against hMPV A1 and B1 subtypes in human serum, in order to create a suitable, cell-based, semi-quantitative method that could be applied to large-scale serological studies with high throughput.

To standardize and validate this EMN assay, we carried out analytical procedures according to the International Council of Harmonization guidelines in order to provide reliable, accurate and reproducible results^18^.

Of note, some hindrances in set-up and validation of the method were evaluated and should be highlighted here.

The main drawback in our assay validation lay in the lack of an established Standard for hMPV. Although International Standards could facilitate the standardization of serological assays, through the efficient comparison of data from different laboratories^26,27^, no WHO International Reference material for hMPV was commercially available. Thus, a PCR-positive human serum for hMPV was chosen as a positive control in the validation experiments.

Several studies have shown that hMPV had been circulating in the human population for many decades before its first isolation in 2001^1,28^. This is supported by a strong serological evidence of prior hMPV infection in adult populations worldwide. Interestingly, hMPV infections induce a poor immunological memory response, which is the reason why re-infections can occur throughout life. In addition, giving that hMPV is predominantly found in young children (under 5 years of age), it is quite difficult to find subjects with no previous exposure to the pathogen. In this context, we investigated the viral dose to be used in the EMN assay, in order to set-up a highly sensitive method. Results demonstrated a significant relationship between the viral dose used and the nAbs titers elicited, confirming the high sensitivity of the assay at all viral doses tested. However, we deemed that 2000 TCID50 ml^−1^ was the right infective dose, because of the high hMPV infection rate in humans.

Along with the analytical validation, an in-depth clinical evaluation is required before a new technique is implemented in clinical trials. In addition to conducting ICH guideline-based validation experiments, we screened a cohort of serum samples collected from adults in the EMN assay; this confirmed the homogeneous spread of the virus in the population and suggested the potential existence of a cross-protection between A1 and B1 strains.

The same panel of human samples was also tested by means of ELISA, in order to measure levels of total antibodies against the target virus. Interestingly, a significative difference emerged between antibody titers against hMPV-B1 Fusion protein (F0) and antibody titers against hMPV-B1 G protein. This finding highlights the strong immunogenicity of the F protein, which promoted a marked humoral response in comparison with the moderate amount of antibodies detectable against the G protein. Unfortunately, no hMPV-A1 Fusion protein (F0) was commercially available for use in the ELISA test. Our preliminary data obtained from the analysis only of hMPV-A1 G protein suggest that the two subtypes behave in a similar way. Further support for this conviction is provided by the fact that the primary antibody used in the validated EMN assay specifically binds F protein residues that are the same in both strains.

In addition, we observed a close concordance between the results yielded by the two different methodologies (EMN assay and ELISA) when antibody titers against F protein were compared, thus confirming that the F protein is the most attractive target for both serological assays and therapeutic approaches.

To conclude, all the analytical procedures reported in this paper demonstrate the suitability of the EMN assay for the semi-quantitative detection of serum-nAbs against hMPV A1 and B1 strains.

Then, this study paves the way to an accelerated A2 and B2 subtypes characterization. Future efforts will focus on expanding the applicability of this validated approach and exploring new opportunities for a polyvalent vaccine strategy.

## Materials and methods

### Cell and virus propagation

Vero E6 cells (American Type Culture Collection, ATCC #CRL-1586) were grown in high-glucose Dulbecco’s Modified Eagle’s Medium (DMEM) (Euroclone) supplemented with 10% v/v fetal bovine serum (FBS) (Euroclone), 2mM L-glutamine, 100 units ml^−1^ penicillin, and 100 μg ml^−1^ streptomycin (P/S) (Gibco). Cells were maintained at 37 °C in a humified 5% CO_2_ environment and passed every 3–4 days. hMPV-A1 (NL/1/00) and hMPV-B1 (NL/1/99) were purchased from the European Virus Archive—Global portal (EVAg) and provided by the Erasmus University Medical Centre Rotterdam (EVAg Ref-SKU: 011V-00930 and 011V-01003, respectively).

Viral propagation was performed in 175 cm^2^ tissue-culture flasks pre-seeded with 50 ml of Vero E6 cells (1.5 × 10^5^ cells ml^−1^) diluted in DMEM 10% FBS. After 18–24 h at 37 °C in 5% CO_2_, flasks were washed with 1X sterile Dulbecco’s phosphate buffered saline (DPBS1X) (Gibco) and then inoculated with the virus at a multiplicity of infection (MOI) of 0.001. For the inoculum, stock virus was diluted in serum-free DMEM supplemented with 5µg ml^−1^ trypsin (TPCK-treated) (Sigma-Aldrich). Flasks were incubated with the virus for 1 h at 35 °C in 5% CO_2_, then filled with DMEM 2% FBS and 5µg ml^−1^ of trypsin and incubated at 35 °C in 5% CO_2_. Cells were monitored daily until 70–80% of cytopathic effect (CPE) was observed. Harvest was performed by collecting the supernatant and scraping the remaining cell layer from the flasks. The viral solution was centrifuged at 296*g* for 5 min at 4 °C; 5 ml of supernatant and the cell pellet underwent three freeze–thaw cycles, performed by placing the vial on dry-ice (30 s), then at 37 °C (30 s) and subsequently vortexing (30 s). Upon completion, the vial was re-centrifuged to isolate cells, and the supernatant obtained was added to the previously collected viral solution, mixed gently, aliquoted and stored at -80 °C in the presence of 20% sucrose.

### Serum samples

To evaluate the sensitivity of the EMN assay, a total of 16 commercially available human serum samples (Panel F and Panel D, Clinisciences) were screened for the hMPV-A1 strain only.

The experimental protocol for the validation of the EMN assay used a human serum sample positive for hMPV by PCR as a positive homologous control, and a depleted human serum lacking IgA/IgG/IgM (Sigma‐Aldrich) as a negative control. To assess specificity, the positive sample was tested in parallel with the commercially available sheep hyperimmune sera used as heterologous samples: Influenza anti-A/Victoria/2570/2019-like (H1N1) HA Serum 21/120 (NIBSC); Influenza anti-A/Cambodia/e0826360/2020-Like (H3N2) HA Serum 21/118 (NIBSC); Influenza anti-B/Washington/02/2019-like (B-Victoria lineage) HA Serum 19/318 (NIBSC); Influenza anti-B/Phuket/3073/2013-like HA serum (B-Yamagata lineage) 19/322 (NIBSC). Also, an haemolyzed sample (INT-01H, Sun Diagnostic) was used for specificity experiments.

In order to test the reliability of the assay, a total of 105 human serum samples were randomly selected from the samples available. The samples had been collected in the Apulia region (Southern Italy). The research protocol was approved by the Ethics Committee of the University Hospital of Bari (n. 7622, prot. N. 0023599|09|03|2023). The serum survey was conducted in accordance with ethical principles (Declaration of Helsinki), and written informed consent was obtained from all the participants. Samples have been fully anonymized before testing. Serum samples were heat-inactivated by incubation at 56 °C for 30 min before testing.

### ELISA-based microneutralization assay

The EMN assays were performed in 96-well, flat-bottomed, tissue-culture, microtiter plates. DMEM 1% FBS supplemented with 3μg ml^−1^ of trypsin was the complete medium used for the test. Regarding the virus concentration, 25 or 100 or 300 TCID50 (50% tissue culture infectious dose) per well (corresponding to 500, 2000 and 6000 TCID50 ml^−1^, respectively) of virus was used in the set-up experiments to determine the most appropriate infective dose; 100 TCID50 per well was the viral dose used in all the validation tests.

Serum samples were seeded at a final concentration of 1:10 and serial two-fold dilutions were performed. The appropriate dose of virus was then added to each well and the serum-virus mixture was incubated for 1 h at 35 °C, 5% CO_2_. At the end of the incubation time, 100 μl per well were transferred from dilution plate to cell plate pre-seeded at 24 h with 2.0 × 10^5^ cells ml^−1^ in DMEM 2% FBS and incubated at 35 °C, 5% CO_2_.

After 24 h (for hMPV-A1) and 48 h (for hMPV-B1) of incubation, the plates were washed twice with DPBS1X. Fixation was performed by adding 100 μl per well of a cold 80% v/v solution of Acetone (Sigma-Aldrich) in DPBS1X and incubating the plates for 10 min at room temperature (RT); the plates were then emptied and air-dried before the ELISA read-out. Plates were washed three times by means of an automatic plate washer using 300 μl per well of wash buffer, a DPBS1X solution with 0.3% of Tween20 (Sigma‐Aldrich). The primary antibody, Anti-prefusion viral F protein DS7 (Absolute Antibody), was added (100 μl per well) and plates were incubated for 1 h at RT. Subsequently, the plates were washed three times, as previously, and the secondary antibody, Goat Anti-Mouse IgG (H + L)-HRP Conjugate (Bio-Rad), was added (100 µl per well) for an incubation time of 1 h at RT in the dark. Both primary and secondary antibodies were diluted in a 1:1000 ratio by using the antibody diluent, a 5% non-fat dried milk (NFDM)(AppliChem) solution in wash buffer. Next, the plates were rinsed six times with 300 μl per well of wash buffer, and 100 μl of the substrate solution 3,3′,5,5′-Tetramethylbenzidine (TMB) (Sigma-Aldrich) was added to each well. The plates were incubated for 20 min at RT in the dark. The reaction was stopped by adding 100 μl per well of 0,5 M Hydrochloric Acid (Fisher Scientific) and the plates were read at an optical density of 450nm by means of a SpectraMax plate-reader (Medical Device). Softmax Software (GxP Compliance) was used for data collection.

### Validation of ELISA-based microneutralization assay

The EMN assay was validated by testing samples in four different analytical sessions run by two operators over 2 days and obtaining three reportable (RP) values. For each of the three replicate measurements, the GMT was calculated. Specificity and robustness were demonstrated by testing samples in two independent runs performed by two operators.

#### Dilutional linearity

To assessment of linearity, the PCR-positive control was tested in a two-fold dilution scheme in which at least one dilution had a titer below the lower limit of quantitation of the assay, starting from a dilution of 1:10. Thus, the range analyzed was the following: 1:10, 1:20, 1:40, 1:80, 1:160, 1:320, 1:640, 1:1280. The abovementioned sample dilutions were tested in one repetition per plate, in three different plates by two operators on Days 1 and 2. The parameter was evaluated by examining the relationship between the base-2 logarithm of the GMT (observed titers) and the base-2 logarithm of the serum dilutions across the factorial design. The R^2^, y-intercept and slope of the regression line were calculated and reported.

#### Relative accuracy

Data for the evaluation of relative accuracy correspond to the RP values obtained from linearity tests. According to ICH guideline Q2(R2)^20^, the accuracy can be tested by using either a conventional true value or an accepted reference value. The GMT of the expected values was calculated from the GMT of the results obtained from the neat sample, and by dividing this value by the corresponding factor of the two-fold serial dilution. Relative accuracy was evaluated by calculating the percentage of recovery on the GMT of the RP values and the expected (true) titer by applying the formula: 100 × (GMT observed/GMT expected).

#### Precision

Precision was assessed by using the results obtained in the linearity tests and considering three different aspects: repeatability, intermediate precision, and FV.

Repeatability, or intra-run variability, is the variation expected across replicates under the same operating conditions over a short period of time^20^.

Intermediate precision is determined from the total variance component. It represents the variations expected between different laboratories, and those due to the impact of different factors, such as days, environmental conditions, operators, and equipment.

FV represents the variation expected across GMT results yielded by multiple replicates in routine testing. Two independent runs, each of which consisting of one replicate, were considered for FV calculation.

#### Limit of quantitation and range

The LLOQ and ULOQ were determined as the lower and upper 95% CI of the observed GMT of the lowest and highest sample concentrations analyzed with acceptable linearity, accuracy and precision.

#### Specificity

Specificity was assessed by testing the hMPV PCR-positive sample in parallel with commercial anti-heterologous samples. The positive sample had to show a four-fold difference from the anti-heterologous samples tested.

#### Robustness

Robustness is the capacity of an analytical procedure to remain unaffected by small but deliberate variations in method parameters. Two critical conditions were evaluated: cell seeding concentration and incubation time of the virus-serum mixture.

### ELISA

96-well plates (Nunc, Maxi-Sorp) were coated with either 1 µg ml^−1^ of hMPV strain A glycoprotein G Protein (His Tag) (Sino Biological), or 1 µg ml^−1^ of hMPV strain B glycoprotein G Protein (His Tag) (Sino Biological) or 1 µg ml^−1^ of hMPV B Fusion Glycoprotein F0, His-Tag (HEK293) (Native Antigen).

Samples were diluted 1:100 in 5% NFDM solution in TBS (Tris-buffered saline with 0.05% Tween 20, Thermo Scientific). After this step of dilution, for the sessions involving the glycoprotein G of both strains, 10 more steps of two-fold dilution, ranging from 1:100 to 1:51,200, were applied to all samples. In the case of Glycoprotein F0, instead, the range of measurement was extended to 20 dilution points from 1:100 to 1: 52,428,800. After 1 h in blocking solution (5% NFDM in TBS-Tween solution) at RT with shaking, the plates were washed; 100 µl of each serum dilution was then added to the coated plates, which were incubated for 1 h at RT with shaking. After another washing step, polyclonal goat anti-Human IgG-Fc (Bethyl Laboratories) HRP-conjugated antibody was added, and the plates were incubated for 1 h at RT with shaking. After a washing step, TMB substrate was added, and the plates were incubated in the dark at RT for 20 min. The reaction was stopped with 0,5 M Hydrochloric Acid and read at 450nm by means of SpectraMax plate-reader (Medical Device). SoftMax Pro Software – GxP edition 7.1.2 was used for data collection.

### Statistics and reproducibility

All the graphs and the statistical analyses were generated and calculated by GraphPad Prism software version 10.2.3. Statistical analyses performed for validation experiments were executed on Excel 365 Apps for business and R version 4.3.1 software. Correlation between EMN assay and ELISA results was determined by Spearman’s rank correlation coefficient analysis. Further statistical details on the individual experiments are provided in the respective methods section and figure legend.

## Supplementary Information

Supplementary Information.

## Data Availability

The data that support the findings of this study available within the paper and its supplementary information files.
