# Supplementary material for: Design and validation of a semi-quantitative microneutralization assay for human Metapneumovirus A1 and B1 subtypes
Source: Sci Rep. 2025 Apr 4;15:11614. doi: 10.1038/s41598-025-96567-6 (PMC11971440; doi:10.1038/s41598-025-96567-6)
Supplement: Supplementary file 1 — Supplementary Information. [file 41598_2025_96567_MOESM1_ESM.docx]

**Design and validation of a semi-quantitative microneutralization assay for human Metapneumovirus A1 and B1 subtypes**

Giulia Riolo^1,*^, Valentina Biagini^1^, Noemi Guerrini^1,2^, Giulia Roscia^1^, Roberta Antonelli^1^, Ginevra Giglioli^1^, Maria Alfreda Stincarelli^1^, Pietro Piu^1^, Carolina Bonifazi^1^, Simona De Grazia^3^, Mariangela Pizzo^3^, Piero Lovreglio^4^, Angela Stufano^4^, Claudia Maria Trombetta^5,6^, Alessandro Manenti^1^, Emanuele Montomoli^1,5,6^, Francesca Dapporto^1,^.

1. VisMederi S.r.l., Siena, Italy
2. Department of Life Sciences, University of Siena, Siena, Italy
3. Department of Health Promotion, Mother and Child Care, Internal Medicine and Medical Specialties "G. D'Alessandro", University of Palermo, Palermo, Italy
4. Interdisciplinary Department of Medicine, Section of Occupational Medicine, University of Bari, Bari, Italy
5. Department of Molecular and Developmental Medicine, University of Siena, Siena, Italy
6. VaepiX, Joint Research Laboratory, University of Siena, Siena, Italy

*Corresponding author

**Supplementary items**
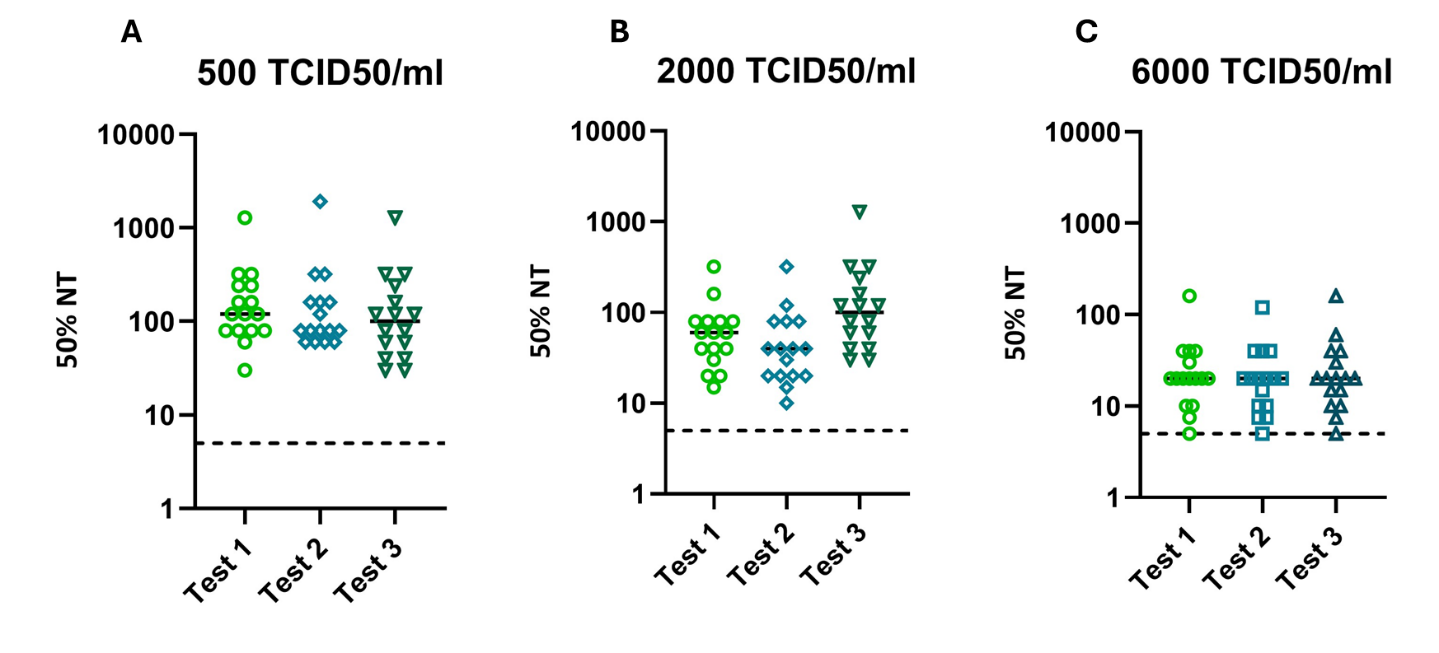


**Supplementary Figure 1.** Viral dose selection and evaluation of neutralization assay reproducibility. Each dot/square/triangle represents the GMT of a single serum sample tested in duplicate in each test at different viral doses 500 (**A**) 2000 (**B**) and 6000 (**C**) TCID50 ml^-1^ for hMPV-A1; *y axis* shows 50% NT expressed as the reciprocal of the serum dilution. Lower limit of detection is set at 5, which is half of the reciprocal of the first serum dilution in plates (dashed black line). Cut-off for the calculation of the 50% NT was determined by applying the formula: *cut-off = ((average VC wells- average of CC wells)/2) + average of CC wells*. GMT: geometric mean titer; NT: neutralizing titer; CC: cell control; VC: viral control.

| Dilutional Linearity hMPV-A1 | | | | | | | | | | | | |
| --- | --- | --- | --- | --- | --- | --- | --- | --- | --- | --- | --- | --- |
| Fold Dilution | **Day 1** | | | | | | **Day 2** | | | | | |
|  | **Op1**  **Rep1** | **Op1**  **Rep2** | **Op1**  **Rep3** | **Op2**  **Rep1** | **Op2**  **Rep2** | **Op2**  **Rep3** | **Op1**  **Rep1** | **Op1**  **Rep2** | **Op1**  **Rep3** | **Op2**  **Rep1** | **Op2**  **Rep2** | **Op2**  **Rep3** |
| 1 | 640 | 640 | 640 | 640 | 640 | 640 | 640 | 640 | 640 | 640 | 640 | 640 |
| 2 | 320 | 320 | 320 | 320 | 320 | 320 | 320 | 320 | 320 | 160 | 320 | 320 |
| 4 | 160 | 160 | 80 | 80 | 160 | 80 | 80 | 160 | 160 | 80 | 80 | 80 |
| 8 | 80 | 80 | 40 | 80 | 80 | 80 | 40 | 40 | 80 | 80 | 40 | 80 |
| 16 | 40 | 40 | 20 | 40 | 40 | 40 | 40 | 40 | 40 | 40 | 40 | 40 |
| 32 | 20 | 20 | 20 | 20 | 20 | 20 | 20 | 10 | 20 | 20 | 20 | 20 |
| 64 | 10 | 10 | 10 | 10 | 10 | 10 | 10 | 10 | 10 | 10 | 10 | 10 |
| 128 | 5 | 5 | 5 | 10 | 10 | 10 | 5 | 5 | 5 | 5 | 5 | 5 |

| Dilutional Linearity hMPV-B1 | | | | | | | | | | | | |
| --- | --- | --- | --- | --- | --- | --- | --- | --- | --- | --- | --- | --- |
| Fold Dilution | **Day 1** | | | | | | **Day 2** | | | | | |
|  | **Op1**  **Rep1** | **Op1**  **Rep2** | **Op1**  **Rep3** | **Op2**  **Rep1** | **Op2**  **Rep2** | **Op2**  **Rep3** | **Op1**  **Rep1** | **Op1**  **Rep2** | **Op1**  **Rep3** | **Op2**  **Rep1** | **Op2**  **Rep2** | **Op2**  **Rep3** |
| 1 | 640 | 640 | 320 | 640 | 640 | 640 | 640 | 640 | 640 | 1280 | 1280 | 1280 |
| 2 | 320 | 320 | 320 | 320 | 320 | 320 | 320 | 320 | 320 | 640 | 320 | 320 |
| 4 | 160 | 160 | 160 | 160 | 320 | 160 | 160 | 160 | 160 | 160 | 160 | 160 |
| 8 | 80 | 80 | 40 | 80 | 160 | 80 | 160 | 80 | 80 | 80 | 80 | 80 |
| 16 | 40 | 40 | 40 | 40 | 80 | 40 | 40 | 40 | 40 | 40 | 40 | 40 |
| 32 | 20 | 20 | 10 | 20 | 20 | 20 | 40 | 20 | 40 | 20 | 20 | 20 |
| 64 | 10 | 10 | 10 | 20 | 20 | 10 | 20 | 20 | 20 | 10 | 10 | 10 |
| 128 | 5 | 5 | 5 | 5 | 5 | 5 | 10 | 10 | 10 | 5 | 5 | 5 |

**Supplementary Table 1**. Neutralizing titers obtained in Dilutional linearity experiments for hMPV-A1 and hMPV-B1. Data results from four different analytical sessions run by two operators over two days. Each sample has been tested in one repetition per plate, in three different plates, obtaining three replicates per session. Op: operator; Rep: replicate.

| Relative accuracy | | | | | | | | |
| --- | --- | --- | --- | --- | --- | --- | --- | --- |
| Fold Dilution | **hMPV-A1** | | | | **hMPV-B1** | | | |
|  | **Observed GMT** | **Expected GMT** | **Relative accuracy** |  | **Observed GMT** | **Expected GMT** | **Relative accuracy** |  |
| 1 | 640 | 640 | 100% | **PASSED** | 718 | 718 | 100% | **PASSED** |
| 2 | 302 | 320 | 94% | **PASSED** | 339 | 359 | 94% | **PASSED** |
| 4 | 107 | 160 | 67% | **PASSED** | 170 | 180 | 94% | **PASSED** |
| 8 | 63 | 80 | 79% | **PASSED** | 85 | 90 | 94% | **PASSED** |
| 16 | 38 | 40 | 95% | **PASSED** | 42 | 45 | 93% | **PASSED** |
| 32 | 19 | 20 | 95% | **PASSED** | 21 | 22 | 95% | **PASSED** |
| 64 | 10 | 10 | 100% | **PASSED** | 13 | 11 | 118% | **PASSED** |
| 128 | 6 | 5 | 120% | *Not valid* | 6 | 6 | 100% | *Not valid* |

**Supplementary Table 2.** Relative accuracy for hMPV-A1 and hMPV-B1. Valid dilutions that exhibit a ratio of observed to expected GMT of between 50% and 200% are labelled as “PASSED”. When the observed GMT is lower than 10, dilution results to be “Not valid”. GMT: geometric mean titer.

**Supplementary Data Analysis 1.** Variance components and precision assessment.

To partition and quantify the contribution to the total observed variation of various factors present during ELISA-based microneutralization analysis, we employed a linear mixed-effects model. These factors included the operators and the days of experimentation. To enhance precision, we conducted assessments on the base-2 logarithmic transformation of the measured titers. The mixed-effects model was implemented for each sample, incorporating Operator nested within Day as a random effect. This approach allows us to account for variability due to different operators performing the analysis on the same day, as well as day-to-day variability. The model was implemented using the lme4 package in R with the following code:

```R lmer(log2Titer ~ (1|DAY/OP), control = lmerControl(optimizer = "nloptwrap", optCtrl = list(algorithm = "NLOPT_LN_BOBYQA")))```

The Bound Optimization BY Quadratic Approximation (BOBYQA) algorithm was chosen as the optimizing function in the control argument of the function.

The total observed variance $\sigma_{T}^{2}$consists of two components:

1. $\sigma_{T}^{2}=\sigma_{IR}^{2}+\sigma_{R}^{2}$

$\sigma_{IR}^{2}$, which is the inter-run variance component and $\sigma_{R}^{2}$, which is the residual variance component. This total observed variance can be used to estimate the Intermediate Precision (%GCV= geometric coefficient of variation) for each sample, while the residual variance can be utilized to assess the intra-run effect (%GCV) as follows:

1. Intermediate Precision % GCV = $\left( 2^{\sqrt{\sigma_{T}^{2}}}-1 \right).100$
2. Intra-run Precision % GCV = $\left( 2^{\sqrt{\sigma_{R}^{2}}}-1 \right).100$

The expected variability between reported results when multiple replicates are combined into a final geometric mean titer (GMT) is known as format variability (%GCV). As recommended in USP <1033>, assessment of assay precision is not required to be undertaken employing the routine assay format (runs, replicates and reported GMT) rather the validation experiments should be designed so the components of the assay variability can be estimated.

The inter-run variability was estimated from the random effects of Operator nested in Day, and the intra-run variability was given directly from the residual component. After estimating the inter-run and intra-run variance components, we estimated the variability in routine GMT results produced using certain assay formats. The proposed assay format (number of runs and replicates per run) where an acceptable Format Variability (FV) for all samples was observed can be implemented for routine testing. Format Variability (%GCV) was estimated as follows:

1. Format Variability % GCV = $\left( 2^{\sqrt{\frac{\sigma_{IR}^{2}}{k}+\frac{\sigma_{R}^{2}}{nk}}}-1 \right).100$

where *n* is the number of replicate sets and *k* the number of runs produced in routine testing. When significant variability is detected between different runs or within the same run during validation testing, adjustments may be made to the number of runs or replicates. This is to ensure that the variability in GMT results falls within acceptable limits during routine assay testing. The acceptable level of variability is defined such that the expected GMT result for any sample should not differ by more than ±2-fold from the anticipated titre level.

The linear mixed-effects model yields an ANOVA type III table where one can estimate the variance components (in terms of %GCV): intermediate precision, intra-run precision and FV (equations 2-4).

The predicted variability for k independent runs, with n number of replicate sets of the test preparation within a run, is given by the above equation 4. We set n=1 and k=2, because in the routine testing, the runs are independent of each other. Noticeably, chapter <1033> of the USP guideline also defines the critical fold difference (CFD), as “the size of the fold difference that can be distinguished between samples tested in the bioassay”. This quantity turns out to be the pivotal quantity whereby the acceptance criteria for FV and IP can be deduced. We can calculate the CFD directly from the format variability as follows:

1. $CFD=2^{q_{\alpha}\sqrt{\frac{\sigma_{IR}^{2}}{k}+\frac{\sigma_{R}^{2}}{nk}}}$

where q_α_ is the quantile of interest (from the t-distribution with alpha = 1 % and infinite degrees of freedom, which is well and properly approximated by the standard normal percentile z_α_ = 2.326348).

From equation 4 we obtain

1. $\sqrt{\frac{\sigma_{IR}^{2}}{k}+\frac{\sigma_{R}^{2}}{nk}}=log\left( 1+FV \right)$

therefore, we can rewrite CFD:

1. $CFD= 2^{{log\left( 1+FV \right)}^{z_{\alpha}}}=\left( 1+FV \right)^{z_{\alpha}}$

Of course, if we use the base-2 exponential instead of the natural exponential *e*, we will get the same result by applying the log_2_ transform.

In these Variance Component Analysis-based precision assessments, the acceptance criteria for the repeatability, the intermediate precision and the format variability have been set equal to 65.5%, 129% and 81.5%, respectively.

*Repeatability acceptance criterion*

The threshold for the repeatability is determined with reference to previous results.

*Intermediate Precision acceptance criterion*

When taking the base-2 log-transform, since the desired CFD is 4:

1. $CFD=2^{z_{\alpha}\cdot\sqrt{\frac{\sigma_{IR}^{2}}{k}+\frac{\sigma_{R}^{2}}{nk}}}= 4$

then we have

1. $\frac{\sigma_{IR}^{2}}{k}+\frac{\sigma_{R}^{2}}{n\cdot k}=\left( \frac{2}{z_{\alpha}} \right)^{2}=0.7391=\left[ {log}_{2}\left( 1+FV \right) \right]^{2}$

which is equivalent to the radicand of the FV. The radicand of IP can be expressed in terms of FV, that is

1. $\sigma_{IR}^{2}+\sigma_{R}^{2}=n\cdot k\cdot\left( \frac{\sigma_{IR}^{2}}{k}+\frac{\sigma_{R}^{2}}{n\cdot k} \right)-\left( n-1 \right)\cdot\sigma_{IR}^{2}=n\cdot k\cdot\left( \frac{2}{z_{\alpha}} \right)^{2}-\left( n-1 \right)\cdot\sigma_{IR}^{2}$

A test design will be adopted where n=1 and k=2 therefore the above result can be simplified as

1. $\sigma_{IR}^{2}+\sigma_{R}^{2}= 2\cdot\left( \frac{2}{z_{\alpha}} \right)^{2}$

Finally, threshold for the intermediate precision parameter will be calculated

1. ${IP}_{max}=\left( 2^{\sqrt{2\cdot\left( \frac{2}{z_{\alpha}} \right)^{2}}}-1 \right)\cdot100=\left( 2^{\frac{2}{z_{\alpha}}\cdot\sqrt{2}}-1 \right)\cdot100$

When rounding to one decimal place (in equation 13, the approximation is expressed using the *round* function of the r-software), the following is obtained:

1. ${IP}_{max}=2^{round\left( \frac{2}{z_{\alpha}}*sqrt\left( 2 \right),digits=1 \right)}-1=129.7\%$

Finally, by truncating this value to the integer part, the maximum value of intermediate precision shrinks to 129%. This is equivalent to setting an acceptability criterion that is prudentially more restrictive (approximately -3%) than the theoretical one.

*Format Variability acceptance criterion*

The direct link between the CFD and the FV has been introduced above. From equation 7, for a given maximum CFD equal to 4 and z_α_ = 2.326348, the maximum FV can be calculated backward.

1. $4=\left( 1+FV \right)^{2.326348}\to{log}_{2}\left( 1+FV \right)=\frac{2}{2.326348}\to FV=4^{{2.326348}^{-1}}-1=0.8146819$

That is approximated to 81.5% in terms of GCV%. The acceptance criterion for format variability is established such that, if adhered to, the variability among multiple replicates of reported mean results will not allow any expected outcome in a long series to exceed four times the anticipated result. For instance, if the expected titre is 10, no reported mean should exceed a titre of 40. In prospective validation studies, if the assay's format variability is deemed unacceptable, it is recommended to increase the number of replicates, days, operators, and other parameters to ensure acceptable variability prior to testing clinical trial samples. This four-fold approach aligns with the calculation of seroconversion in clinical statistics regarding relative accuracy and linearity. When using the log_2_ titre scale and assuming a normal distribution of log-transformed data, the maximum allowable imprecision of reported results will determine the acceptable level of imprecision to prevent a titre of 10 from being misclassified as positive at 40 or higher. If a titre of 10 is expected then the maximum Geometric Standard Deviation (GSD) can be determined that ensures, in a long series of measurements, that 99.0% of data points are less than a titre of 40:

1. $10\cdot{GSD}^{2.326348}\leq40\therefore GSD \leq1.815$

that is equivalent to %GCV ≤ 81.5%.

| Specificity | hMPV-A1 | hMPV-B1 |
| --- | --- | --- |
| hMPV PCR positive - HP-HS | 640 | 1280 |
| Influenza anti-A/Victoria/2570/2019-like (H1N1) HA Serum 21/120 - HP-HET | 5 | 10 |
| Influenza anti-A/Cambodia/e0826360/2020-Like (H3N2) HA Serum 21/118 - HP-HET | 5 | 10 |
| Influenza anti-B/Washington/02/2019-like (B-Victoria lineage) HA Serum 19/318 - HP-HET | 5 | 10 |
| Influenza anti-B/Phuket/3073/2013-like HA serum (B-Yamagata lineage) 19/322 - HP-HET | 5 | 5 |
| Sun Diagnostic cod. INT-01H - HP-HET | 5 | 5 |
| Negative control serum | 5 | 10 |

**Supplementary Table 3.** Specificity results for hMPV-A1 and hMPV-B1. Neutralizing titers correspond to the GMT retrieved from two independent tests, where samples have been tested in duplicate in each test. GMT: geometric mean titer.

|  | Cell seeding concentration | | Serum-virus incubation time | |
| --- | --- | --- | --- | --- |
|  | **SD** | **GVC (%)** | **SD** | **GVC (%)** |
| hMPV-A1 | 0.2 | 25.32 | 0.2 | 24.04 |
| hMPV-B1 | 0.3 | 36.62 | 0.3 | 29.85 |

**Supplementary Table 4.** Robustness results for hMPV-A1 and hMPV-B1. SD: standard deviation; GCV: geometric coefficient of variation.
